# An Ideal Scheme of Co-Operation

**Published:** 1892-05-07

**Authors:** 


### The Institutional Workshop.

### HOSPITAL ADMINISTRATION.

### AN IDEAL SCHEME OF CO-OPERATION.

A conference of workers of the Metropolitan Hospital Saturday Fund was held in the Lecture Hall of the City Temple on the 27th ult., to hear an address by Mr. Henry C. Burdett, on an ideal scheme of co-operation between doctors, artisans, and hospitals, with a view to strengthening the Hospital Saturday Fund in London. Mr. Reginald B. Acland, Chairman of the Council of the Fund, presided, and was supported by Mr. H. N. Hamilton-Hoare, the Treasurer.

The Chairman, in introducing Mr. Burdett to the meeting, said that he felt sure that Mr. Burdett, in his past criticisms on the working of the Fund, was actuated solely by a desire to see the interests of the Fund advanced in every way possible.

#### Preliminary Considerations.

Mr. Burdett said that before coming to the scheme proper which he wished to place before them, it was necessary to recognise fully all the interests and issues involved in an accurate consideration of the hospital question. These interests were those of the doctors, the artisans, the hospitals, and the ordinary subscriber to the hospitals. Doctors were a link between the artisans and the hospitals, and in any consideration of such a question as the relief of the sick poor in hospitals and institutions, the interests of the doctors must be kept well in mind. First, then, no system of medical relief in any country should be so organised as to unduly interfere with the means which an honourable man engaged in the practice of medicine had of obtaining a livelihood if he should happen to reside in a poor district. Admitting that it was evident such a man must be paid for his services, and must have a certain number of patients. Next it was essential that the wage-earning classes—the artisans—should receive the maximum of medical care when sick, and as good medical attendance in their own homes as any other class of the community. For that very reason it was necessary that they should have members of the medical profession who would devote their lives to attendance upon the sick in the relatively poorer districts of all large cities. It might be said that good medical attendance could only be secured at the hospitals, and, indeed, this contention was often put forward. His answer to that was, that he had been through most of the poorer districts of London and had found practising there medical men with the highest qualifications that the best medical colleges could confer. If that were so, under the present circumstances, they might be sure that if medical men were properly encouraged many more would throw in their lot with their poorer brethren, because numbers preferred to practise in poor districts, knowing that there they could do the greatest good. That brought him to the hospitals, and he would premise that they were not for the charitable relief of the well-to-do of any class whatever. Their duty was primarily to provide medical relief for those unable to obtain it otherwise. After having done that, they might provide relief for others who should pay for their treatment according to their means. The great mistake made in the English system of hospital administration was in placing the onus of proof of ineligibility for free relief on the hospitals. This should be reversed, each man who required relief being obliged—as in America—to prove that he was a fit person to occupy a free bed, or else to prove what amount he could afford to pay for his treatment. This was by the way, but he mentioned it because he felt it was a cure for the great cry of abuse of hospitals, which one heard so much of in the present day. Coming back to the subject, the question of the ordinary subscriber and his ticket had finally to be considered. Subscribers to hospitals seemed to think that unless their tickets were in demand, and were all used up in the year, they were being defrauded of a right. This was a wrong view to take of the matter, as surely every man who gave a subscription to a charity did not give it with a view to getting a return for his money; rather, he should take care that for every ticket he used he gave an additional sum, equal to the cost of the patient's treatment, to the funds of the hospital.

#### An Ideal Scheme of Co-operation.

So much for the general considerations. Keeping these in mind, what was the best scheme which, while it kept all the interests involved in view, would present a solution of the question of relief? It was evident that the work must commence with the hospitals as the centre or pivot on which everything turned. The reason of the great success of the Hospital Sunday Fund was that it had inside each institution voluntary workers—the clergy, churchwardens, and congregations—who collected large sums for the hospitals at a small cost. The Hospital Saturday Fund had exactly the same helpers if they were only properly organised, namely, the employers, the foremen, and the artisans, and the help of all these must be secured in the first place for any scheme which was to be really successful. Through the foremen, weekly subscriptions of a half-penny, or a penny, or twopence, would be organised from every employé in the workshop, and these contributions would be put into a box. Added to this would be a contribution from the employer, assessed by him on the basis of his profits. Letters of introduction to doctors and hospitals would be provided for the foremen, and whenever a man in their workshop or one of his family fell sick, he would be given first of all a letter to the doctor in his district. If the case was a minor one, the doctor would attend to it, recovering his fee from the box. Should, however, the case be a serious one, one which required nursing and other advantages not obtainable at the patient's home, the patient would be given a letter of introduction to the hospital he might select, and would be received within its walls. The hospital in such a case would keep a record of the nature of the case, and how long it remained in the hospital, and would render an account to the workshop it came from, receiving in return a sum equal to its cost taken from the box. That was a very simple plan, its great merit being that in it they had a workable system, one which safe-guarded the interests of all, and one which left to the artisan a further privilege—the privilege of bestowing a free gift upon the hospitals. This latter would be arrived at thus:—On Hospital Saturday each year the box would be opened, and the amount contained therein, after deducting a certain percentage for medical appliances, would be sent to the central body, the Hospital Saturday Fund (upon which the workshop would be represented) as a gift from the artisans to be distributed among the hospitals. Besides representation on the central committee the contributions from a workshop, for work done, would entitle it to representation in the hospital which ministered to the wants of that workshop.

#### What the Hospital Saturday Fund Should Not Be.

In conclusion, Mr. Burdett gave his views on what the Hospital Saturday Fund should not be, what it should be, and what it might be. It should not be, he said (1) A central organization collecting money to be doled out in exchange for privileges, *i.e.*, governorships and tickets; (2) an expensive agency for collecting money; that was to say, the justification of a central fund depended, as a matter of business, really upon the amount for which it was able to raise the sum distributed amongst the hospitals. The Metropolitan Hospital Saturday Fund was now, of course, in its expensive stage. Suppose a man bought land and intended to devote it to building purposes. He first of all had to put in the sewers and roads and fences. That initial stage was the most expensive, and that was the stage the Fund had now reached. If, therefore, they found that they were now obliged to use 20 per cent. of the amount collected in working expenses, well and good, its supporters must have patience, and wait. If, however, at the end of five or six years it was found that the expenses were still the same and had not been reduced in any way, then it would be time to raise the question.—Have you justified your existence ? and (3) Not an in gatherer of funds from any but working-class sources ? The working man was proud of his class, and if this was to be his movement then let it be his movement alone. Despite this, and despite his personal dislike of them, he was not going to say that they were not to have street collections, for in London such collections brought in a sum of £5,000, but he did think it would be a good thing if they were cut off altogether from Hospital Saturday.

### What the Hospital Saturday Fund Should Be.

Now came the question of what the Hospital Saturday Fund should be. It should be (1) representative of and in touch with all workshops and manufactories; (2) the treasury for the free gifts of the working classes to the hospitals; (3) a check upon the lessening of gifts from other classes. That was to say that the Hospital Saturday Fund should set a limit on the contributions of the working classes as otherwise other people would be sure, as they found the need of the hospitals grew less, to diminish their subscriptions to them. What the working class contribution should amount to in London he thought might fairly be calculated on the basis of what was done at Birmingham. There the annual expenditure of the hospitals was roughly about £42,000. That of London was some £683,000 or fifteen times as much in the latter as in the former city. Birmingham working men contributed £10,000 a-year towards this expenditure, and therefore the working classes of London should contribute fifteen times that sum, *i.e.*, £150,000 to the support of the hospitals in their midst; and (4) Be affiliated to or co-operating with the Hospital Sunday Fund on this basis: Let each collect and distribute its own money, but both co-operate to find out the best way in which this money can be distributed.

### What the Hospital Saturday Fund Might Be.

What Hospital Saturday might be! Well it might be (1), the articulate voice of the artisans bringing to the notice of hospital managers the requirements of the working-classes; (2), the machinery to gather up the free gifts of the working classes for the relief of those less fortunately circumstanced than themselves; and (3), the connecting link between the workshop, the hospital, and the individual workman.

### THE DISCUSSION.

Mr. J. G. Lemon did not see how if there was not sufficient in the box of a particular workshop to do this, the doctor would be paid for his services under Mr. Burdett's scheme. In all businesses bad debts were contracted, and it seemed to him such a system would be a most fruitful source of bad debts.

Mr. Hill believed that if Mr. Burdett would only have the patience he counselled he would find the Metropolitan Hospital Saturday Fund sooner or latter become the ideal scheme he had fore-shadowed. He agreed with Mr. Burdett that the working man demanded a *quid pro quo* as regards his contributions to the hospitals, but Mr. Burdett seemed to suggest that the money should go into the pockets of the doctors on the principle that doctors must live. Precisely; everyone must live, but each man must try to do the best for himself, so that if the working man thought that he could get better and cheaper treatment on the co-operative principle, *i. e.*, by going to the hospitals than he could through private enterprise; *i. e.*, by going to the general practitioner, why he necessarily will go to the cheaper place.

Mr. R. N. Hamilton-Hoare, also held that the payment of doctors must be supplementary to the welfare of the people. He was astonished that Mr. Burdett had omitted all mention of the work done by the provident dispensaries in London, as the cases which under his scheme would go to the doctors could very well be treated at the dispensaries. Under the box scheme, too, the workshops would lose the power given them by combination in the Hospital Saturday Fund, and the power of a particular hospital would become paramount in the workshop to which that hospital ministered.

Mr. Baker's opinion was that the best doctors were specialists, and they could only be seen by the working-man at the hospitals.

Mr. Bunn (Secretary of the Fund) explained that the reason the Hospital Sunday Fund was enabled to raise a large sum at so small a cost compared with the Saturday Fund was that the contributions were received in much larger amounts at a time, and that they were sent in free of charge. In the Saturday Fund a collecting sheet had to be printed, to be sent by post to the collector, to be returned by him with perhaps a few shillings on it. This amount had then to be booked, and a receipt had to be made out and posted to the collector. He objected to the box scheme on the ground that in London the amount collected in a workshop would certainly not be sufficient to meet the calls upon it. It was only the system of averages which enables the Hospital Saturday Fund to succeed. Practically, the Hospital Saturday Fund were doing as a central body what Mr. Burdett wished to do locally. They had the co-operation of the foremen, the workmen, and the employers, who helped in collecting subscriptions.

Mr. Webster (organising secretary) thought the scheme propounded by Mr. Burdett would be difficult in London owing to the circumstances being totally different. When he came to London nothing astonished him more than the size of the workshops. In the North of England if he got a workshop to give a collection, he obtained from £100 to £200, but in London he had to visit 150 or so workshops before he could get the same amount. As to the amount contributed by the working classes in the provinces, no comparatively equal sum would ever be collected in London.

The Chairman was of opinion that the Metropolitan Hospital Saturday Fund was pretty much what Mr. Burdett had said an ideal Hospital Saturday Fund should be. It was not what he had said it should not be, and it was trying to be the first three things he said it might be. One of the great objects which he wished to see carried out was co-operation with the Hospital Sunday Fund.

Mr. Burdett in reply said the question of the payment of doctors had not been apparently understood. He was not present as an advocate on behalf of the doctors, but all he wanted to bring home was that if they complained of the medical attendance which was near their homes at the present time, the way to improve its quality was to pay for it, and to pay ready money for it. As to the question of boxes a very slight modification would make the new system meet all the objections made to it. Make the Central Fund the box, and out of it pay for all the medical treatment obtained the sum it actually costs the hospital.

The meeting concluded with votes of thanks to Mr. Burdett and the Chairman.

